# Decreasing cytosolic translation is beneficial to yeast and human Tafazzin-deficient cells

**DOI:** 10.15698/mic2018.05.629

**Published:** 2018-02-18

**Authors:** Maxence de Taffin de Tilques, Jean-Paul Lasserre, François Godard, Elodie Sardin, Marine Bouhier, Marina Le Guedard, Roza Kucharczyk, Patrice X. Petit, Eric Testet, Jean-Paul di Rago, Déborah Tribouillard-Tanvier

**Affiliations:** 1 Institut de Biochimie et Génétique Cellulaires, CNRS UMR 5095, Université de Bordeaux, 1 rue Camille Saint-Saëns, 33077 Bordeaux cedex, France.; 2 Laboratoire de Biogenèse Membranaire, CNRS UMR 5200, Université de Bordeaux, INRA Bordeaux Aquitaine, Villenave d'Ornon, France.; 3 LEB Aquitaine Transfert-ADERA, FR-33883 Villenave d'Ornon, Cedex, France.; 4 Institute of Biochemistry and Biophysics, Polish Academy of Sciences, Warsaw, Poland.; 5 CNRS FR3636 Fédération de recherché en Neuroscience, Université Paris-Descartes, 45, rue des Saints-Pères, 75006 Paris, France.

**Keywords:** mitochondrial disease, oxidative phosphorylation, Barth syndrome, cytosolic protein synthesis, cycloheximide; cardiolipin remodeling

## Abstract

Cardiolipin (CL) optimizes diverse mitochondrial processes, including oxidative phosphorylation (OXPHOS). To function properly, CL needs to be unsaturated, which requires the acyltransferase Tafazzin (TAZ). Loss-of-function mutations in the TAZ gene are responsible for the Barth syndrome (BTHS), a rare X-linked cardiomyopathy, presumably because of a diminished OXPHOS capacity. Herein we show that a partial inhibition of cytosolic protein synthesis, either chemically with the use of cycloheximide or by specific genetic mutations, fully restores biogenesis and the activity of the oxidative phosphorylation system in a yeast BTHS model (*taz1*Δ). Interestingly, the defaults in CL were not suppressed, indicating that they are not primarily responsible for the OXPHOS deficiency in *taz1*Δ yeast. Low concentrations of cycloheximide in the picomolar range were beneficial to TAZ-deficient HeLa cells, as evidenced by the recovery of a good proliferative capacity. These findings reveal that a diminished capacity of CL remodeling deficient cells to preserve protein homeostasis is likely an important factor contributing to the pathogenesis of BTHS. This in turn, identifies cytosolic translation as a potential therapeutic target for the treatment of this disease.

## INTRODUCTION

The Barth Syndrome (BTHS) is a rare X-linked recessive mitochondrial disorder that is characterized by cardiac and skeletal myopathies, growth retardation, hypocholesterolemia, increased urine levels of 3-methylglutaconic acid and high susceptibility to bacterial infections, due to cyclic neutropenia 1,2,3. This disease is caused by mutations in Tafazzin, a mitochondrial protein involved in the remodeling of cardiolipin (CL). This phospholipid is mainly found in mitochondria, 4,5,6,7,8,9,10 where it optimizes numerous processes including oxidative phosphorylation (OXPHOS) 11,12,13, fusion 14, fission 15,16, protein import 17,18, iron-sulfur cluster biogenesis 19, mitophagy 20,21,22,23, apoptosis 7,23,24,25,26,27,28 and the transport of metabolites across the mitochondrial inner membrane 6,17,29,30,31,32,33,34,35,36. Tafazzin is an acyltransferase required for the maintenance of unsaturated carbon-carbon bonds in CL fatty acyl chains 1,37,38,39,40,41. Loss-of-function mutations in Tafazzin lead to reduced levels of unsaturated CL and the accumulation of CL species with an incomplete set of fatty acyl chains (such as monolysocardiolipin, MLCL) 42,43,44. This in turn results in multiple mitochondrial alterations that ultimately compromise the OXPHOS capacity 24,45,46,47,48.

Simple model organisms such as *Saccharomyces cerevisiae* or baker's yeast are an important resource for the study of mitochondrial diseases. Mitochondria from this single-celled fungus and humans show many similarities 49,50,51,52,53. Being easily amenable to genetic manipulation of mitochondrial function 54,55, and owing to the ability of yeast to survive the loss of oxidative phosphorylation; yeast models of human mitochondrial diseases can be easily created and kept alive when provided with fermentable substrates 56,57. The common respiratory growth defect of these models enables large-scale screening of genetic and pharmacological suppressors 57,58,59. Yeast has in this way already pointed to several potential druggable therapeutic intervention points, such as the oxodicarboxylic acid carrier 60 and mitochondrial protein import 61, among others.

Herein we report that reducing cytosolic protein synthesis preserves OXPHOS in CL remodeling deficient yeast and improves the growth rate and viability of human HeLa cells lacking Tafazzin. This study sheds new light on the pathogenesis of BTHS and identifies cytosolic protein synthesis as a potential intervention point for the treatment of the disease.

## RESULTS

### Decreasing cytosolic protein synthesis improves respiratory growth of *taz1*Δ yeast

**Figure 1:**
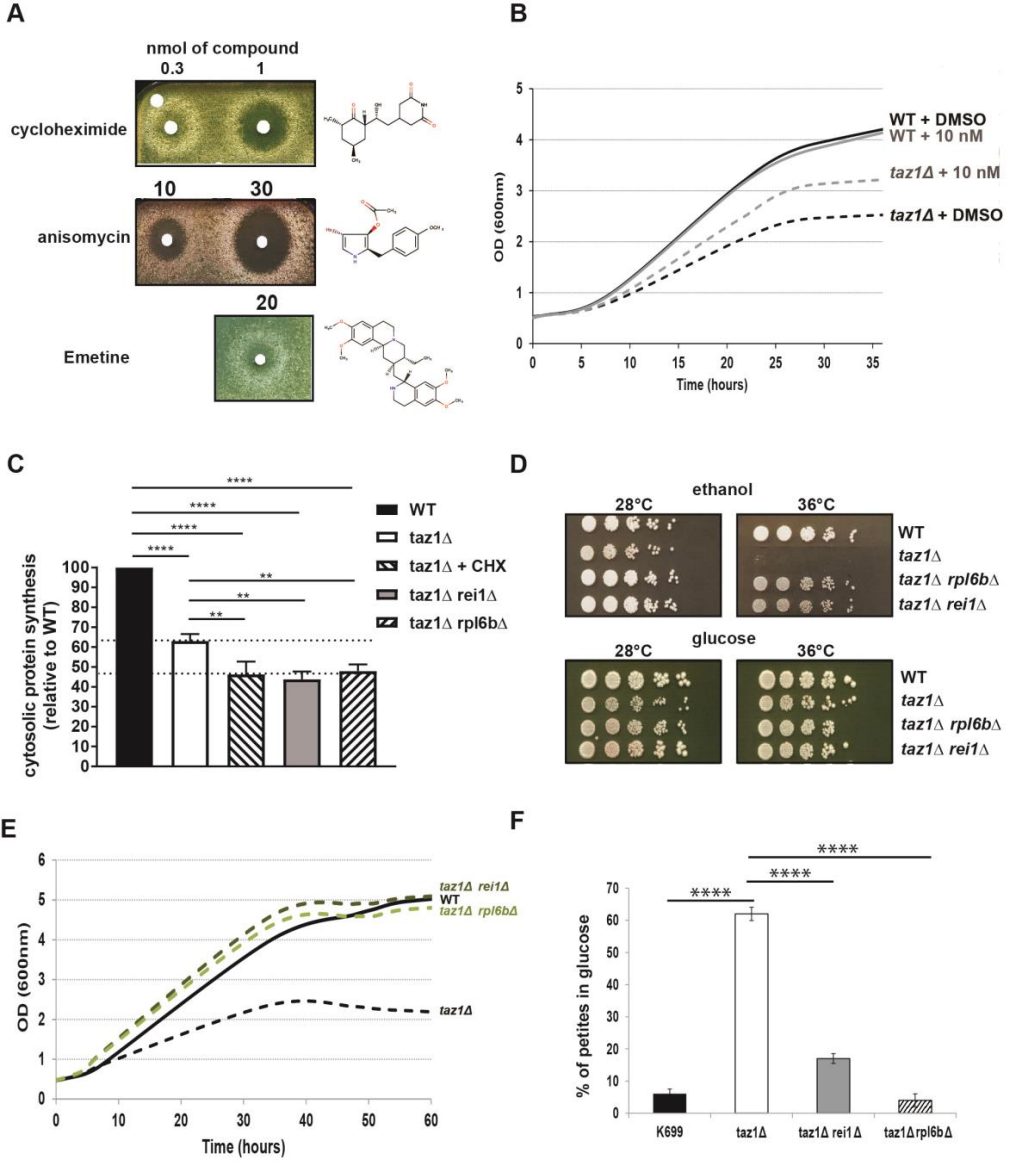
FIGURE 1: Partially decreasing cytosolic translation in Taffazin-deficient (*taz1*Δ) yeast improves respiration-dependent growth and mtDNA maintenance*.* (**A)** *taz1*Δ yeast cells were spread as dense layers onto rich ethanol solid media and then exposed to sterile filters spotted with cycloheximide, anisomycin or emetine (dissolved in DMSO). The plates were scanned after 5 days of incubation at 36°C. The filter at the top left was spotted with DMSO alone to provide a negative control. **(B)** Determination in liquid cultures of CHX concentrations that optimally rescue *taz1*Δ yeast. Complete synthetic media (CSM) containing 0.5% galactose + 2% ethanol supplemented or not with CHX at the indicated concentrations were inoculated with WT and *taz1*Δ cells pre-grown in CSM containing 2% glucose at 28°C. The cultures were performed at 36°C and cells densities (OD_600nm_) taken over a period of 36 hours. **(C) **Rate of cytosolic protein synthesis. Total proteins and mitochondrial proteins were labeled with a mixture of [^35^S]-methionine and [^35^S]-cysteine for 20 min in whole cells from wild type, *taz1*Δ *rei1*Δ*, taz1Δ rpl6bΔ *and *taz1*Δ yeast grown for 24 hours in rich 0.5% galactose + 2% ethanol at 36°C, and *taz1*Δ cells grown in the same conditions in presence of 10 nM cycloheximide (CHX). After the labeling reactions, total protein extracts were prepared and separated by SDS-PAGE on a 12% polyacrylamide gel (75 µg per lane). The gels were dried and analyzed with a PhosphorImager. Quantification was performed using Image J. Data are expressed in % relative to the WT (n=3). The shown data are cytosolic protein synthesis rates (total minus mitochondrial protein synthesis rates). Statistical analysis was done with Tukey’s test (*P<0.05; **P<0.01; ***P<0.001; ****P<0.0001). **(D)** Genetic ablation of *REI1* (*rei1Δ*) or *RPL6B* (*rpl6bΔ*) improves respiratory growth of *taz1*Δ yeast. WT, *taz1*Δ, *taz1*Δ* rei1*Δ* and* *taz1Δ rpl6bΔ *cells freshly grown at 28°C in rich glucose were serially diluted and spotted onto rich ethanol and glucose plates. The plates were scanned after 4 days of incubation at the indicated temperature. **(E) **Growth of WT, *taz1*Δ, *taz1*Δ* rei1*Δ* and* *taz1Δ rpl6bΔ *strains in liquid complete synthetic media containing 0.5% galactose + 2% ethanol at 36°C. The cultures were inoculated with cells grown in CSM containing 2% glucose at 28°C. The cultures were performed at 36°C and cell densities (OD_600nm_) taken over a period of 60 hours. **(F) **Genetic ablation of *REI1* (*rei1*Δ) or *RPL6B* (*rpl6b*Δ) in *taz1*Δ yeast preserves mtDNA maintenance. Proportions of ρ^-^/ρ^0^ cells produced in glucose cultures at 28°C of strains WT, *taz1*Δ, *taz1*Δ* rei*Δ, and *taz1Δ rpl6bΔ *were determined using the procedure described in 60 (n=3). Data are expressed in % relative to the WT and were statistically analyzed using Tukey’s test (*P<0.05; **P<0.01; ***P<0.001).

We 60 and others 62 showed that yeast cells lacking the gene encoding Taz1p (*taz1*Δ) grow poorly on respiratory carbon sources at 36°C, compared to the wild-type (WT) *TAZ1^+^* strain. Using a drug screening procedure we previously described 58, we found that cytosolic protein synthesis inhibitors such as, cycloheximide (hereafter abbreviated as CHX), anisomycin and emetine suppressed this phenotype in a dose-dependent manner. In the tests shown in Fig. 1A, *taz1*Δ cells freshly grown by fermentation in glucose were spread on plates containing ethanol (respiratory carbon source) and then exposed to paper disks spotted with the drugs dissolved in DMSO. After five days of incubation at 36°C, halos of enhanced growth appeared around the filters, whereas DMSO alone had no effect. In this assay the compounds diffused into the medium, explaining why growth was improved only at some distance around the filters, below which it was totally absent due to a too high concentration of the protein synthesis inhibitors. CHX was active at 20-30 fold lower concentrations compared to anisomycin and emetine (Fig. 1A). The optimal rescuing concentration range of CHX was determined by growth tests in liquid media containing 2% ethanol and 0.5% galactose, at 36°C. After consumption of the galactose, which is a fermentable substrate, growth of *taz1*Δ yeast was much less efficient compared to the WT, owing to its failure to properly express mitochondrial function (Fig. 1B). The best growth improvement of *taz1*Δ yeast was observed in the presence of 10 nM CHX. At this concentration, growth of the wild type was unaffected (Fig. 1B). Pulse labeling of proteins with S^35^-methionine and S^35^-cysteine revealed that the rate of cytosolic protein synthesis was decreased by about 50% in* taz1*Δ yeast grown in the presence of 10 nM CHX, in comparison to the WT (Fig. 1C). Interestingly, cytosolic translation was already decreased in the mutant grown in the absence of the drug by about 35%, possibly as a means to attenuate a protein stress induced by a lack in CL remodeling (see below).

If CHX is a well-known inhibitor of cytosolic translation, one cannot exclude that it has other effects in cells that could be responsible for the improved respiratory growth of *taz1*Δ yeast. We therefore tested the effects on *taz1*Δ yeast of null mutations in the genes *REI1* and *RPL6B *that are known to partially inhibit cytosolic protein synthesis by 20% and 30% respectively 63,64. The double mutants *taz1*Δ *rei1*Δ and *taz1Δ rpl6bΔ *grew efficiently on respiratory carbon sources (Fig. 1D, E), and showed a 50% drop in the rate of protein synthesis (Fig. 1C). These data confirmed that the beneficial effect of CHX in *taz1*Δ yeast resulted from a decreased rate of protein synthesis.

### Decreasing cytosolic protein synthesis improves mtDNA maintenance in *taz1*Δyeast

We previously showed that *taz1*Δ yeast grown by fermentation at 28°C, i.e. in conditions where the presence of functional mtDNA is not indispensable, has an increased propensity to produce ρ^-^/ρ^0^ cells issued from large deletions in the mitochondrial genome (60% *vs* 5% in the WT) 60. The double mutants *taz1*Δ *rei1*Δ and *taz1Δ rpl6bΔ *produced five to ten times less ρ^-^/ρ^0^ cells than *taz1*Δ yeast in glucose cultures (Fig. 1F). Thus, partially decreasing cytosolic translation preserves a proper maintenance of mtDNA in CL deficient yeast cells.

#### Reducing cytosolic translation does not restore CL remodeling in *taz1*Δ yeast

**Figure 2:**
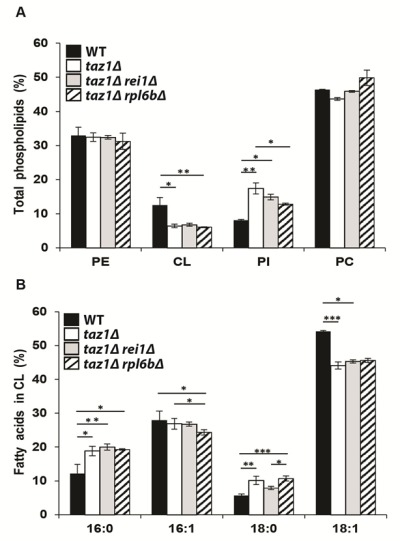
FIGURE 2: Genetic ablation of *REI1* or *RPL6B* in *taz1*Δ yeast does not restore cardiolipin remodeling. Lipids were extracted from mitochondria isolated from WT (black bars), *taz1*Δ (open bars), *taz1*Δ* rei1*Δ (grey bars) and *taz1*Δ* rpl6b*Δ (striped bars) cells grown in CSM 0.5% galactose + 2% ethanol at 36°C until a density of 2-3 OD_600nm_. **(A)** Relative contents of PE (phosphatidylethanolamine), CL (cardiolipin), PI (phosphatidylinositol) and PC (phosphatidylcholine) within each strain. **(B)** Relative fatty acid chain composition of CL within each strain (16:0, palmitic acid; 16:1, palmitoleic acid; 18:0, stearic acid; 18:1: oleic acid). Statistical analysis was done with Kruskal-Wallis test followed by Dunn’s multiple comparison test (*P<0.05; **P<0.01; ***P<0.001). Data are expressed as mean ± s.d. (n=4). The data for WT and *taz1*Δ strains were reported previously 60.

As reported 39,60,62, mitochondria from *taz1*Δ yeast, compared to those from the WT, have 50% less CL, a 2-fold higher content in phosphatidylinositol (PI), whereas phosphatidylethanolamine (PE) and phosphatidylcholine (PC) accumulated normally (Fig. 2A). Additionally, the remaining CL species are less unsaturated as suggested by the decreased levels in oleic acid chains (C18:1) and increased stearic (C18:0) and palmitic (C16:0) groups compared to CL molecules extracted from the WT (Fig. 2B). Strains *taz1*Δ *rei1*Δ and *taz1Δ rpl6bΔ *showed very similar phospholipid profiles (Fig. 2A, B), indicating that mitochondrial function recovery in *taz1*Δ yeast upon partial inhibition of cytosolic translation did not result from an enhanced production of mature CL species.

#### Partially decreasing cytosolic protein synthesis fully restores OXPHOS in *taz1*Δyeast

**Figure 3:**
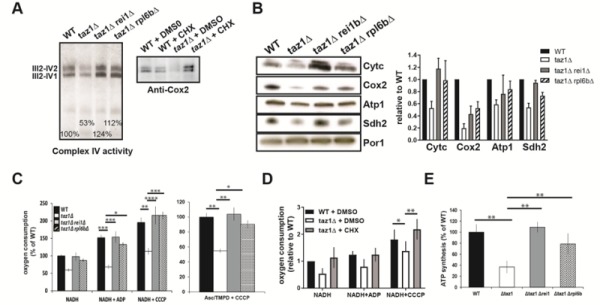
FIGURE 3: Partially decreasing cytosolic translation preserves oxidative phosphorylation in *taz1*Δ yeast. The experiments here described were performed using mitochondria isolated from cells grown for 24 hours at 36°C in CSM containing 0.5% galactose + 2% ethanol, supplemented or not as indicated with 10 nM CHX, until a density of 2-3 OD_600nm_. **(A, B)** Steady-state levels of proteins involved in the transfer of electrons to oxygen. **(A)** Proteins were extracted from the mitochondrial samples using 2 g digitonin per g of proteins. The supercomplexes III_2_-IV_2_ and III_2_-IV_1_ were revealed by the complex IV activity after separation by CN-PAGE or by western blot with antibodies against Cox2 in BN-PAGE gels. **(B) **Left panel. Total mitochondrial protein samples were resolved by SDS-PAGE (50 μg per lane) and probed with antibodies against the indicated proteins. The shown gels are representative of at least 3 experiments. Right panel. Quantification using ImageJ software. Levels of Cytc, Cox2, Atp1 and Sdh2 are normalized to Por1p and expressed relative to WT. **(C)** Genetic ablation of *REI1* or *RPL6B* in *taz1*Δ yeast preserves mitochondrial respiration. On the left are the rates of oxygen consumption from NADH (4 mM) alone (state 4), after further addition (150 μM) of ADP (state 3) or CCCP (4 μM) (uncoupled respiration). The data are expressed in % of WT state 4 respiration (mean ± s.d, n=4). On the right are the oxygen consumption rates from electrons delivered directly to complex IV by ascorbate 12.5 mM/TMPD 1.4 mM in the presence of CCCP. Data are expressed relative to the WT (mean ± s.d, n=4). **(D)** Mitochondrial respiration is preserved in *taz1*Δ yeast grown in the presence of 10 nM CHX. NADH was used as the electron donor, as described in panel C (n=4). **(E)** ATP synthesis was measured using NADH as a respiratory substrate in the presence of 1 mM ADP. Data are expressed as mean ± s.d. (n=4) relative to the WT. The data for WT and *taz1*Δ strains were reported previously 60.

As we have shown 60, the reduced ability of *taz1*Δ yeast to grow at 36°C in 2% ethanol + 0.5% galactose (shown in Fig. 1B) correlated with a decreased rate of oxygen consumption and diminished levels of key components involved in the transfer of electrons to oxygen, including complexes II-IV and cytochrome *c*. Accumulation of these proteins (Fig. 3A, B) and oxygen consumption measured with either NADH or Ascorbate-TMPD as electron donors were substantially improved after eliminating *REI1* or *RPL6B* (Fig. 3C) or growing *taz1*Δ yeast in the presence of 10 nM CHX (Fig. 3D). Consequently, the rate of mitochondrial ATP synthesis was restored to almost normal levels (Fig. 3E).

**Figure 4:**
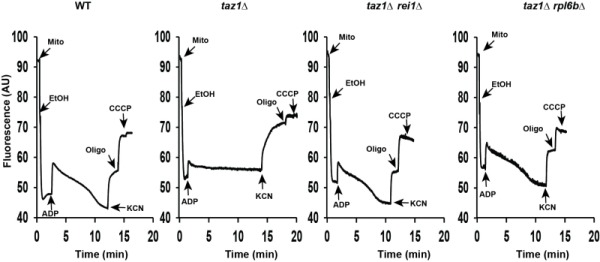
FIGURE 4: Mitochondrial membrane potential. Variations in mitochondrial ΔΨ were monitored by fluorescence quenching of Rhodamine 123, using intact, osmotically-protected, mitochondria isolated from WT, *taz1*Δ, *taz1*Δ* rei1*Δ and *taz1*Δ* rpl6b*Δ cells grown in CSM containing 0.5% galactose + 2% ethanol at 36°C until a density of 2-3 OD_600nm_. The additions were 75 μM ADP, 0.5 μg/ml Rhodamine 123, 75 μg/ml mitochondrial proteins (Mito), 10 μl ethanol (EtOH), 2 mM potassium cyanide (KCN), 4 μM CCCP (carbonyl cyanide-m-chlorophenyl hydrazone) and 4 μg/ml oligomycin (oligo). The shown tracings are representative of four experimental trials. The data for WT and *taz1*Δ strains were reported previously 60.

These observations were corroborated by monitoring changes in mitochondrial membrane potential (ΔΨ) with Rhodamine 123. As we showed 60, adding ADP to respiring *taz1*Δ mitochondria did not decrease ΔΨ due to their poor capacity to synthesize ATP. Consistent with their good ability to produce ATP, those from strains *taz1*Δ *rei1*Δ and *taz1Δ rpl6bΔ *efficiently responded to ADP as WT mitochondria (Fig. 4). Furthermore, after a subsequent addition of KCN, to block the respiratory chain, ΔΨ collapsed in one single rapid phase in *taz1*Δ mitochondria, whereas a residual potential was preserved in those from *taz1*Δ *rei1*Δ and *taz1Δ rpl6bΔ *and WT yeasts. This residual potential is due to the pumping of protons by ATP synthase, coupled by the hydrolysis of the ATP that accumulated in the mitochondrial matrix during phosphorylation of the added ADP, as evidenced by the loss of this potential by inhibiting ATP synthase with oligomycin (Fig. 4).

Taken together, these observations demonstrate that partially decreasing cytosolic translation preserves the biogenesis and activity of the OXPHOS system in CL remodeling deficient yeast.

#### Partially decreasing cytosolic protein synthesis suppresses the enhanced production of ROS in taz1Δ yeast

**Figure 5:**
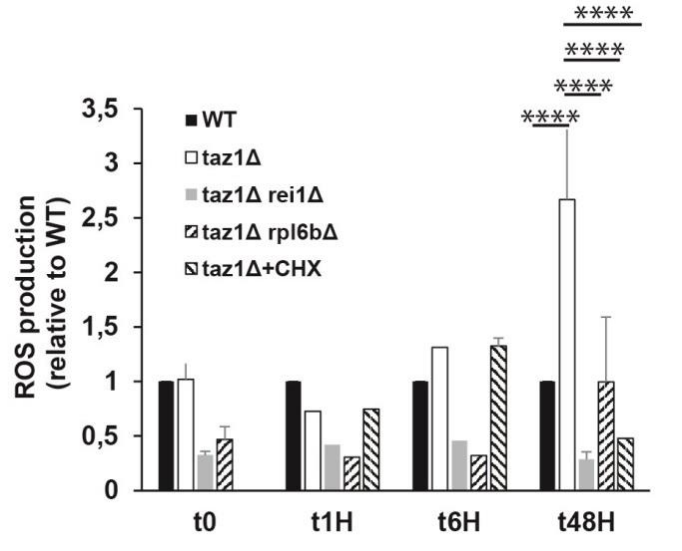
FIGURE 5: Partially decreasing cytosolic translation in *taz1*Δ yeast preserves a normal production of ROS. The cells were grown in CSM containing 0.5% galactose + 2% ethanol at 36°C for 48 hours. At the indicated times, ROS levels were measured by flow cytometry using dihydroethidium as a probe. The data are expressed in % relative to the WT at T0 (n=3). Statistical analysis was done with Tukey’s test (*P<0.05; **P<0.01; ***P<0.001; ****P<0.0001). The data for WT and *taz1*Δ strains were reported previously 60.

Defects in the mitochondrial respiratory chain often result in a higher production/accumulation of reactive oxygen species (ROS) owing to an enhanced diversion of electrons from their normal pathway to oxygen, which was observed in CL remodeling deficient cells 65. Thus, it was expected that *taz1*Δ yeast should produce less ROS after deleting *REI1* or *RPL6B* or during growth in the presence of 10 nM CHX, which was indeed observed (Fig. 5).

### Decreasing cytosolic protein synthesis is also beneficial to tafazzin-deficient human cells

We aimed to understand whether partially decreasing cytosolic translation could also benefit human cells lacking Tafazzin. To this end, we used our previously described HeLa cells, in which the TAZ gene was knocked down by RNA interference (shTaz1) and two control cell lines, shWT1 and shTaz1R in which the expression of TAZ1 was not inhibited. As reported, ShTaz1 poorly accumulates Tafazzin, is defective in CL maturation, has a reduced capacity to associate respiratory chain complexes into ‘respirasomes’, produces abnormally enlarged cells and has a higher content in mitochondria compared to shWT1 and shTaz1R, as do cells from BTHS patients 24,48,66.

**Figure 6:**
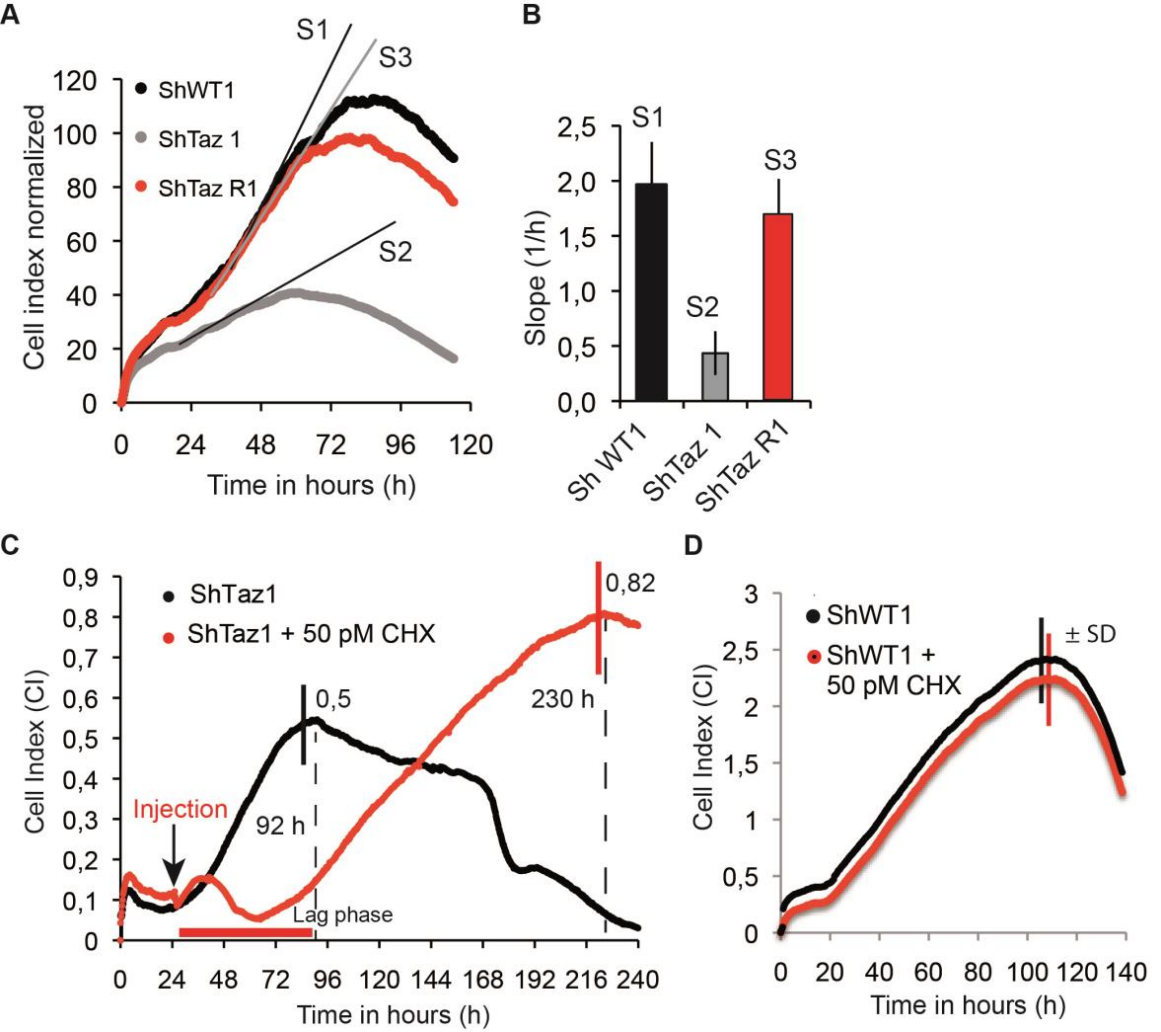
FIGURE 6: CHX improves cell proliferation and viability of human Tafazzin-deficient cells. These experiments used our previously described HeLa cells in which TAZ gene has been knocked down by RNA interference (shTaz1) and two control cell lines, shWT1 and shTaz1R, in which expression of TAZ1 is not inhibited 67. **(A)** Growth curves in 200 µL wells inoculated with 5 000 cells. After reaching a plateau, the cells die and detach from their support. S1, S2 and S3 are the slopes of the proliferation state for each cell lines. **(B)** Relative slopes (1/h) deduced from the proliferation curves shown in panel A. **(C)** xCELLigence recording of ShTaz1 proliferation in absence or presence of CHX at a concentration of 50 pM. CHX was added (black arrow) after a 24-hour adhesion step. The cultures were inoculated with 5 000 cells in 200 μL wells. The optimal Cell Index values are indicated along the curves as well as the time (in hours) taken to reach the inflexion point. The horizontal red bar stands for the 48 hours lag phase induced by CHX. Four independent experiments with 4 wells for each growth condition have been done (16 wells in total for establishing mean values). the ±SD is directly drawn on the top of the curves (in black for ShTaz1 and in red for SgTaz1 + 50 pM CHX). **(D)** xCELLigence recording of ShWT1 proliferation in absence or presence of CHX at a concentration of 50 pM. The cultures were inoculated with 5 000 cells in 200 μL wells. CHX was added before the binding of the cell to the substrate in this case in order to avoid the perturbations induced by the injection of CHX along the trace; the ±SD is directly drawn on the top of the curves (in black for ShWT1 and in red for SgTaz1, there is no significant variation).

Herein we report that ShTaz1 cells proliferate four times slower and die more rapidly, in comparison to ShWT1 and ShTazR1 cells (Fig.6 A, B). We took advantage of these differences to test the capacity of CHX at counteracting the detrimental effects of a lack in CL maturation in human cells. A large beneficial effect was observed at a 50 pM concentration of CHX. In the experiment shown in Fig. 6C, the drug was added 24-25 hours after inoculating 200 µl of media with 5000 cells. CHX induced a 48 h lag phase, after which the cells grew for a long-lasting period of 170 hours, before dying and detaching from their support. In the absence of CHX, death was observed much more rapidly, after 72 hours of continuous growth. At the concentration of 50 pM, CHX had no effect on the proliferation of ShWT1 (Figure 6D). These observations indicate that a partial decrease in cytosolic translation is, as in *taz1*Δ yeast, beneficial to human cells defective in CL remodeling.

## DISCUSSION

While a general inhibition of cytosolic protein synthesis would obviously be detrimental to the cell, our study reveals that a partial (50%) decrease in this activity preserves mtDNA maintenance and the biogenesis and activity of the oxidative phosphorylation (OXPHOS) system in a yeast model of the Barth syndrome, a mitochondrial disease associated to defects in the remodeling of cardiolipin (CL). The decreased mtDNA stability in *taz1*Δ yeast occurred in fermenting (glucose) cultures where the presence of this DNA is not indispensable. With a respiratory carbon source (glycerol), *taz1*Δ yeast cells lacking functional mtDNA were much less abundant owing to their incapacity to proliferate in these conditions. Thus, the respiratory deficiency of *taz1*Δ yeast does not result from a lack in mtDNA.

Importantly, CL remodeling was still deficient in *taz1*Δ cells rescued by a partial inhibition of cytosolic translation. This finding has two important corollaries: (i) the CL species remaining in the mutant (50% vs the WT) are sufficient for a proper biogenesis and functioning of the OXPHOS system, and (ii) the OXPHOS deficit is secondary to some other cellular dysfunction(s) that can be suppressed by decreasing cytosolic translation. At low concentration (50 pM), CHX also improved proliferation of HeLa cells deficient in tafazzin whereas that of WT Hela cells was not modified, which clearly demonstrates that growth improvement resulted from a compensation of the lack in tafazzin. Thus yeast and human CL remodeling deficient cells face similar difficulties that can be attenuated by targeting cytosolic translation. These finding reveal that a diminished capacity of CL remodeling deficient cells to preserve protein homeostasis is likely an important factor contributing to the pathogenesis of the Barth syndrome. This in turn, identifies cytosolic translation as a potential therapeutic target for the treatment of this disease.

Previous work revealed that decreasing cytosolic translation can also rescue yeast models of adPEO (autosomal dominant progressive external ophthalmoplegia) caused by mutations in ANT, a protein that exchanges adenine nucleotides across the mitochondrial inner membrane 68. In addition to a defective exchange of adenine nucleotides, these mutations compromise the impermeability to protons of the inner membrane. Consequently, a sufficient electrical potential cannot be maintained across this mem-brane and this impedes many proteins to reach the organelle. This has deleterious consequences, not only for mitochondria, but also for the cytosol that is then confronted with the over-accumulation and the misfolding of mitochondrial precursor proteins 63. This protein stress, which was termed mitochondrial precursor over-accumulation stress (mPOS), was shown to induce a cellular response, named unfolded protein response activated by mistargeted mitochondrial proteins (UPRam), that is mainly characterized by a decreased rate of cytosolic protein synthesis and a faster rate of protein clearance by the proteasome 68,69. Consistent with these findings, mutations in proteins directly involved in the transport of proteins into mitochondria were found to similarly poison the cytosol with mitochondrial proteins triggering the cell to diminish the production and stimulate the degradation of proteins 69. The significant drop (35%) in cytosolic translation in *taz1*Δ yeast is an indication that a lack in CL remodeling could as well lead to the congestion of the cytosol with miss-localized mitochondrial proteins.

Considering the numerous roles of CL in mitochondria, it might be that a lack in the remodeling of this phospholipid affects the capacity of mitochondria to import proteins, and thereby makes other compartments of the cell more susceptible to protein stress. In support to this hypothesis, loss of CL remodeling was shown to partially compromise the biogenesis of the protein translocase (TOM) and the sorting and assembly machinery (SAM) of the outer mitochondrial membrane 18,70. In another study 36, no evidence was found for a decreased preprotein accumulation *in vitro* with *taz1*-deficient mitochondria. However, because these assays used minute amounts of preproteins it might be difficult to detect in this way a partially diminished protein import capacity.

The recovery of mitochondrial function in *taz1*Δ yeast by a partial decrease of cytosolic translation is in line with a recent study showing that CL has an important role in promoting the induction of a mitochondrial-to-cytosolic stress response (MCSR) that enables the cell to improve protein homeostasis in both compartments 71. Consistently also, it was shown that rapamycin, a specific inhibitor of the mTOR signaling pathway that regulates several extra-mitochondrial cellular pathways among which protein synthesis, robustly enhances survival and attenuates the disease’s progression in a mouse model and patient cells of the Leigh Syndrome, one of the most devastating mitochondrial disorders 72,73.

Clearly, beyond a certain level of mitochondrial damage, the protein stress responsive pathways may not be sufficient. This may explain that in addition to its spontaneous 35% drop in protein synthesis, *taz1*Δ yeast requires a further (15%) decrease in this activity to be effectively rescued. Whether a general protein synthesis inhibition or a reduced production of specific proteins is beneficial to CL remodeling deficient cells is an interesting and important issue. In this respect, it is interesting to note that Gerst *et al*. found that ribosomal protein paralogs specifically modulate translation of mitochondrial precursor proteins 74. Such observations hold promise for the development of more targeted therapeutic approaches with less undesirable side-effects to preserve protein homeostasis in cells poisoned by the over-accumulation in the cytosol of mitochondrial protein precursors.

## MATERIALS AND METHODS

### Growth media

The following media were used for growing yeast: Fermentable YPAD media containing 1% (w/v) yeast extract, 1% (w/v) bacto peptone, 40 mg/L adenine and 2% (v/v) glucose; Respiratory rich media YPAEthanol containing 1% (w/v) yeast extract, 1% (w/v) bacto peptone, 40 mg/L adenine and 2% (v/v) ethanol; Non-fermentable complete synthetic media CSM/gal/ethanol containing 0.17% (w/v) yeast nitrogen base without aminoacids and ammonium sulfate, 0.5% (w/v) ammonium sulfate, 0.5% (w/v) galactose, 2% ethanol and 0.08% (w/v) of a mixture of aminoacids and bases from Formedium. Solid media contained 2% (w/v) agar.

### Construction of strains *taz1Δ rpl6bΔ *and *taz1*Δ *rei1*Δ

These strains were constructed by deleting entirely the open reading frame of *RPL6B or REI1 *with the KanMX marker using a described procedure 75 in *taz1*Δ mutant (*MATa* *ade2-1* *ura3-1* *his3-11*, *15* *trp1-1 leu2-3,112 can1-100,* *taz1::TRP1*). For the construction of the strain *taz1*Δ* rpl6b*Δ, the *KanMX *cassette was amplified using primers pFA6a-Kan: Rpl6b-del-F (CTT TCT TGA ACT TGG AAG AGA AGC AAA TAT ATT CAA CGA A cgg atc ccc ggg tta att aa) and Rpl6b-del-R (CTA TTT TAA ATC ATT TAT AAT TTT TTC AGT TCA AT gaa ttc gag ctc gtt taa ac) (the sequences in capital letters are homologous to the *RPL6B *flanking regions, those in lower case enable amplication of *KanMX*. For the construction of the strain *taz1*Δ* rei1*Δ, the *KanMX *cassette was amplified with pFA6a-Kan: Rei1-del-F (CAT TAG AAG TCA AGA AGA GAG CAT ATC AGT AAC AAT ACG cgg atc ccc ggg tta attaa) and Rei1-del-R (GCG ACA AAA TAC TAA AAA AAG TAG TGC AAA AAG AA gaa ttc gag ctc gtt taa ac). The primers Rpl6b-Fbis (CTG CGC TTC CGT TCA GCA TC), Rpl6b-Rbis (CGA TGA CCT GAT CTT GAA CCC) or, Rei1-Fbis (GTG GTG TAG CTA TTT GTA CAT G), and Rei1-Rbis (CAA CAT CTT CAG TCT TCA GCA GC) were used to verify the deletions of *REI1* and *RPL6B by PCR.*

### Yeast-based drug assay

0.125 OD of exponentially growing cell were homogeneously spread with sterile glass beads on a square Petri dish (12 cm x 12cm) containing solid YPA ethanol medium. Sterile filters were deposited on the plate and spotted with cycloheximide (purchased from Sigma), anisomycin (purchased from Sigma), and emetine (purchased from Sigma) dissolved in DMSO. Growth improvement was assessed after several days of incubation at 36°C.

### Bioenergetics experiments

The mitochondria were prepared by the enzymatic method as described 76. Protein concentrations were determined by the Lowry method 77 in the presence of 5% SDS. Oxygen consumption rates were measured on 75 μg of fresh mitochondria using a Clarke electrode in the respiration buffer (0.65 M mannitol, 0.36 mM ethylene glycol-bis(2-aminoethylether)-N,N,N′,N′-tetraacetic, 5 mM tris-phosphate, 10 mM tris-maleate, pH 6.8) as described 78 (see legend of Fig. 3 for the concentrations of reagents used). Variations in mitochondrial transmembrane potential (ΔΨ) were evaluated in the same respiration buffer, by monitoring the quenching of rhodamine 123 fluorescence (0.5 mg/ml) using a λ_exc_ of 485 nm and a λ_em_ of 525 nm under constant stirring, using a FLX Spectrofluorimeter (SAFAS, Monaco), as described 79. ATP synthesis rates were measured using 75 μg of fresh mitochondria in a 2-ml thermostatically controlled chamber at 28°C in respiration buffer, in the presence of 4 mM NADH and 1mM ADP as described 80. Aliquots were withdrawn from the oxygraph cuvette every 15 seconds and supplemented with 2.5% (w/v) perchloric acid, 8.5 mM EDTA to stop the reaction and then neutralized to pH 6.8 by adding 2N KOH, 0.3 M MOPS. ATP was quantified using a luciferin/luciferase assay (ATPLite kit from Perkin Elmer) on a LKB bioluminometer. The participation of the F_1_F_0_-ATP synthase in ATP production was assessed using the same protocol, in the presence of oligomy¬cin (3 µg/ml).

### BN/CN-PAGE & SDS-PAGE

Blue native BN-PAGE and clear native CN-PAGE experiments were carried out as described 81, using mitochondrial extracts solubilized with digitonin (2 gr per gr protein) run in 3-12% con¬tinuous gradient acrylamide gels. The in-gel complex IV activity was revealed using a solution of Tris 5mM pH 7.4, diaminobenzidine 0.5 mg/ml, cytochrome *c* 0.05 mM. The proteins were also analyzed by Western blotting on poly(vinylidene difluoride) membranes as described 82. Polyclonal antibodies raised against yeast ATP synthase were used at a dilu¬tion of 1:50000 for subunit α (gift from J. Velours, IBGC, Bordeaux, France); 1:10000 for cytochrome *c* (gift from S. Manon, IBGC, Bordeaux, France); 1:5000 for succinate dehydrogenase Sdh2 subunit (gift from C. Dallabona, University of Parma, Italy). Monoclonal antibodies against porin and Cox2 (from Molecular Probes) were used at a dilution of 1:5000. Nitrocellulose membranes were incubated with peroxidase-labeled antibodies (from Promega) at a 1:2500 dilution, and analyzed by electrochemiluminescence. Quantification of the protein signals was performed with the ImageJ software.

### *In vivo* labeling of mitochondrial translation products

The indicated strains were grown to early exponential phase (OD/ml of 2) in 20 ml of rich ethanol/galactose media at 36°C. The cells were harvested by centrifugation and washed twice with a minimum medium containing 2% ethanol and 0,5% galactose, supplemented with histidine, tryptophan, leucine, uracil and adenine (50 mg/liter each). To evaluate total protein synthesis, cells were resuspended in 1 ml of the same medium with the addition of 55 µCi of [^35^S] methionine plus [^35^S] cysteine (Amersham Biosciences) and incubated for 20 min at 36°C. To evaluate mitochondrial protein synthesis the same procedure was followed but before adding [^35^S] methionine plus [^35^S] cysteine, cells were first treated with 7,5 mg/ml cycloheximide during 5 minutes. After the labeling reactions, total protein extracts were prepared and quantified using the Lowry method. The proteins were separated by SDS-PAGE on a 12% polyacrylamide gel, transferred onto a nitrocellulose membrane and analyzed with a PhosphorImager. Quantification of radioactive proteins was performed using the software ImageJ 83.

### Lipid analyses

Mitochondrial lipids were analyzed as described 60. In summary, the lipids were extracted with 2 ml of chloroform/methanol (2:1, v/v). After centrifugation, the organic phase was isolated and the remaining lipids were further extracted twice by adding 2 ml of chloroform to the aqueous phase. The organic phases were pooled and evaporated to dryness. The lipids were then resuspended in chloroform/methanol (2:1, v/v). Respective volumes equivalent to 50 µg of acyl chains were spotted on silica plates, four times for each strain. Polar lipids were separated by one dimensional TLC using chloroform/methanol/1-propanol/methyl acetate/0.25% KCl (10:4:10:10:3.6, by vol.) as a solvent 84. The lipids were located by immersing the plates in a solution of 0.001% (w/v) primuline in PBS, followed by visualization under UV light. The zones of the gel corresponding to PE, CL, PI and PC were then scraped and added to 1 ml of methanol/2.5% H_2_SO_4_ containing 5 *μ*g of heptadecanoic acid methyl ester as a standard. The lipid mixtures were incubated at 80***^◦^***C for 1 h, and 1.5 ml of water and 400 µl of hexane were then added. After centrifugation, the hexane phase containing FAMES (fatty acid methyl esters) was isolated. Separation of FAMES was performed as described 39.

### ROS analysis

Cells at 0.4 OD units were taken from liquid cultures, pelleted in a microcentrifuge, resuspended in 1 ml of phosphate-buffered saline (PBS) containing 50 μM dihydroethidium (DHE; Molecular Probes) and incubated at room temperature for 5 min. Flow cytometry was carried out on a Becton-Dickinson Accuri C6 model flow cytometer. The DHE fluorescence indicated was the direct output of the FL2A (red fluorescence) channel without compensation. A total of 100,000 cells were analyzed for each curve.

### HeLa cells culture and transfection

The cervical carcinoma HeLa cell lines were cultured in DME supplemented with 10% FCS and L- glutamine. Transfection of HeLa cells was performed using Lipofectamine 2000 (Invitrogen). Bcl-xL, shTaz, and shCont stable HeLa cell lines were generated by transfection with pcDNA3/Bcl-xL, pSUPER/shTaz, or pSUPER/shCont, respectively, and selected in G418 67. The revertant shTaz1R cell line was generated by cotransfecting shTaz1 HeLa cells with pLpC vector (carrying a puromycin resistance gene), and pcDNA3/Taz mut and stable clones were selected in the presence of puromycin 67.

### xCELLigence real time cellular proliferation measurements

Experiments were carried out using the xCELLigence RTCA DP instrument (ACEA Biosciences, Ozyme, France) placed in a humidified incubator at 37°C and 5% CO_2_. Cell proliferation and cytotoxicity experiments were performed using 16-well plates (E-plate, Ozyme, Montigny le Bretonneux, France). The microelectrodes attached at the bottom of the wells allowed for impedance-based detection of the attachment, spreading and proliferation of the cells. Initially, 180 μL of cell-free growth medium (10% FBS) was added to the wells. After leaving the devices at room temperature for 30 min, the background impedance for each well was measured. Cells were harvested from exponential phase cultures by a standardized detachment procedure using 0.05% Trypsin-EDTA (Invitrogen). Flow cytometry was used to count the cells and test their viability (FSC versus propidium iodide staining). 5000 or 7500 cells in 20 μl were added in each well. After leaving the plates at room temperature for 30 min to allow early cell attachment, in accordance with the manufacturer’s guidelines, they were locked in the RTCA DP device in the incubator and the impedance value of each well was automatically monitored by the xCELLigence system and expressed as a Cell Index value (CI). Water was added to the space surrounding the wells of the E-plate to avoid interference from evaporation. For proliferation assays, the cells were incubated during 120h for toxicity in growth medium (10% FBS) and CI was monitored every 15 min during the whole duration of the experiment. Four replicates of each conditions were used in each test. After an initial assessment of the concentration of cycloheximide which would be non-toxic to the ShWT1 cells, we used a test of proliferation in presence and absence of low doses of cycloheximide (1 pM to 100 pM) either prior to cell seeding or after an initial adhesion phase and at an early proliferative step (at 24-26 hours). All experiments were conducted over 240 hours. All plots were normalized to the Cell Index.
